# Effect of High-Pressure Processing Pretreatment on the Textural Properties of Cooked *Nuovo Maratelli* Rice

**DOI:** 10.3390/foods13244052

**Published:** 2024-12-15

**Authors:** Cristina Arroqui, Sandra Horvitz, María José Noriega, Idoya Fernández-Pan, Francisco C. Ibañez, Paloma Vírseda

**Affiliations:** Institute for Sustainability & Food Chain Innovation—ISFOOD, Universidad Pública de Navarra, Campus Arrosadia s/n, 31006 Pamplona, Spain; cristina.arroqui@unavarra.es (C.A.); sandra.horvitz@unavarra.es (S.H.); nodo@unavarra.es (M.J.N.); idoya.fernandez@unavarra.es (I.F.-P.); virseda@unavarra.es (P.V.)

**Keywords:** amylose, pressurization, cooking method, microstructure, instrumental texture

## Abstract

*Nuovo Maratelli*, a *japonica* rice with an intermediate amylose content, is suitable for *paella* (a traditional Spanish dish) due to its ability to withstand cooking and absorb flavors. In this study, high-pressure processing (HPP) at 400 and 600 MPa (10 min) was used as a pretreatment to improve the properties of rice cooked by either boiling or microwaving. The microstructure and pasting properties of unpressurized and pressurized rice were examined. Also, the cooking time and cooking kinetics were determined for each cooking method. Overall, the pasting properties of the rice were not impacted by the HPP treatments, but the typical polyhedral form of the rice starch granules was lost, especially at 600 MPa. Cooking times were reduced from 14 and 10 min for unpressurized samples to 12 and 8 min (400 MPa) and 8 and 6 min (600 MPa) for boiling- and microwave-cooked rice, respectively. The rice pretreated at 400 MPa (10 min) and microwaved (8 min) had lower hardness and adhesiveness, which was linked to the release of amylose during cooking. In summary, HPP could be an effective pretreatment for the improvement of the cooking and textural properties of *Nuovo Maratelli* rice, particularly when cooked by microwaving.

## 1. Introduction

The production of rice in Europe may be considered low in comparison with world production. However, its use has a high socio-cultural value, and several Mediterranean countries have developed world-famous dishes based on rice. This is the case of *risotto* in Italy or *paella* in Spain. These dishes are traditionally prepared using *japonica* rice cultivars with a lower amylose content than *indica* rice cultivars, which in turn, contributes to their unique cooking and sensory properties [1]. In the preparation of *paella*, pearled (white-core) *japonica* rice cultivars are employed due to their capacity to withstand cooking and absorb flavors [2]. *Bomba* rice is the preferred cultivar for this dish due to its medium amylose content (18%); however, it presents a low-yielding variety, resulting in a higher cost. Consequently, other cultivars with a lower amylose content (14%) are utilized. Thus, in addition to selecting the most suitable cultivars, different processing technologies, like soaking or high-pressure processing (HPP), can be applied to enhance the culinary and cooking quality of rice [3]. Moreover, in comparison with heat treatments, HPP enhances the integrity of starch granules [4], delays starch retrogradation, which causes texture deterioration during storage [5], and decreases the temperature and enthalpy of grain gelatinization [6]. Nevertheless, research has demonstrated that the outcomes anticipated from the implementation of HPP are contingent upon the specific rice cultivar, particularly its amylose content. The impact of HPP on waxy (non-pearled) and *indica* rice cultivars with elevated amylose content has been extensively investigated. This encompasses starch retrogradation [4,5], which has been demonstrated to vary significantly as a function of the leached amylose content. Additionally, alterations in solvent retention capacity exhibited notable distinctions between floury and waxy rice cultivars [7].

The impact of HPP on the microstructure and textural properties of *japonica* rice varieties has been the focus of many studies. However, most of them were carried out on rice grains from oriental *japonica* cultivars [8,9,10,11]. To date, only one study has been carried out to evaluate the changes in the microstructure of starches from Western *japonica* rice grains after HPP [12]. Furthermore, textural changes in rice grains of these varieties have not yet been examined.

The hypothesis of this study is that an HPP pretreatment may improve the cooking and textural properties of a *japonica* cultivar with similar or slightly higher amylose content than *Bomba* rice. Therefore, the objective was to evaluate the impact of HPP pretreatment (400 and 600 MPa for 10 min) on the textural properties of *Nuovo Maratelli* rice cooked by two methods: boiling and microwaving.

## 2. Materials and Methods

### 2.1. Materials and Experimental Design

A medium-grain *japonica* rice cultivar (*Nuovo Maratelli*) was selected for the present study. Samples were supplied by a local company (Producción Arroz Navarra S.L, Arguedas, Spain) and were stored at ambient temperature in the dark until use.

**Figure 1:**
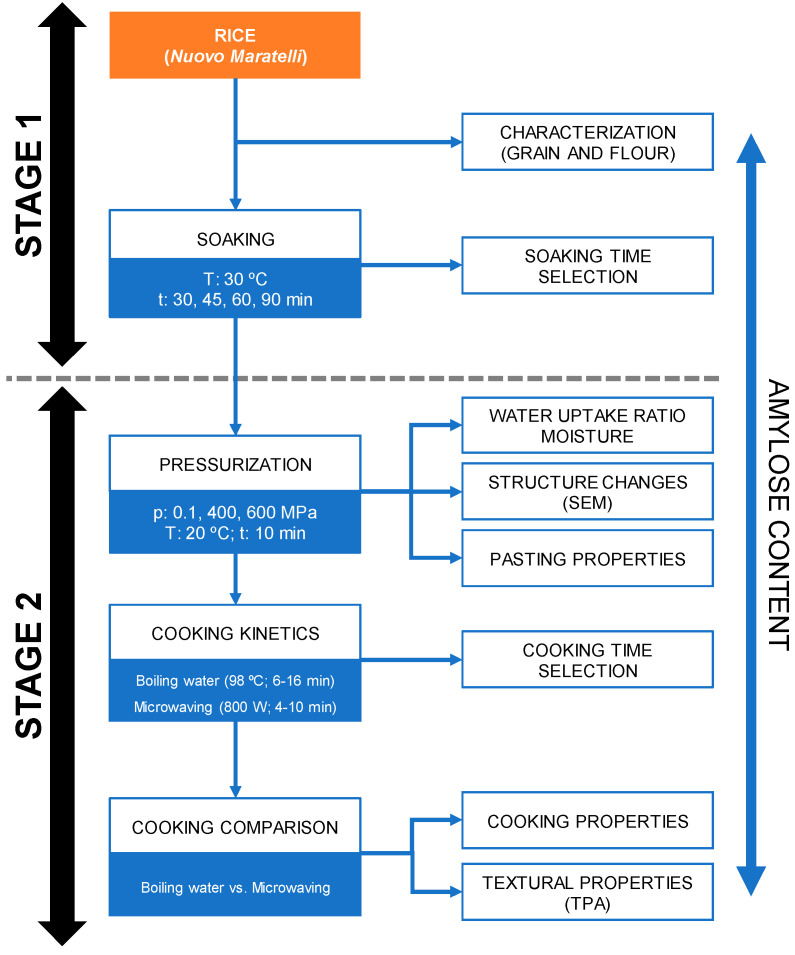
Flowchart detailing the sequence of experimental procedures.

A two-stage approach was used to fulfill the research objectives. Firstly, the optimal hydration level of the rice grain was determined by examining the soaking time. Secondly, the impact of HPP pretreatment on the rice and on the texture and cooking properties of microwave- and boiling-cooked rice was assessed (Figure 1).

### 2.2. Soaking Time and Water Uptake Ratio

The optimal soaking time for achieving the appropriate grain moisture was determined through the method of Rattanamechaiskul et al. [13], with some modifications. The samples (100 g of grain rice and 500 g of water) were placed into a polyamide/polyethylene (PA/PE) 20/70 plastic bag and soaked at 30 °C for 30, 45, 60, and 90 min. The samples were then air-dried for 2 min and weighed. The water uptake ratio of the rice samples (soaked and pressurized) was obtained by dividing the weight of the soaked rice by the initial weight. Once the samples reached a steady state, the optimal soaking time was established. Tests were carried out in triplicate.

### 2.3. High-Hydrostatic Pressure Treatment

A high-pressure unit of 10 L vessel capacity and 800 MPa of maximum pressure (Idus HPP Systems S.L.U., Noain, Spain) was employed for all the experimental assays. Samples of 100 g of rice and 500 mL of osmotized water were placed in a PA/PE 20/70 plastic bag and soaked at 30 °C for the time selected in Section 2.2. The samples were introduced in the HPP equipment at 25 ± 4 °C, and two different levels of pressure were applied, 400 and 600 MPa, for 10 min in both cases. The 10 min treatment time did not include come-up or depressurization times. The pressure-transmitting medium was water, the pressurization rate was 600 MPa/min, and the depressurization was almost instantaneous. The product temperature was registered during processing with a pressure-resistant data logger (LDT200 and Pt-100 thermometer, Leyro Instruments, Barcelona, Spain; Tempmate^®^-B5 data button, Heilbronn, Germany). The collected dataset included both pressure and temperature readings. After treatment, the water was removed, and they were left to dry in air for 2 min before being stored for a maximum of 1 day until further analysis.

### 2.4. Moisture and Amylose Content Determination

The moisture content was estimated following the reference method [14]. For amylose determination, soaked and cooked rice samples were first dried at 40 °C to reduce moisture up to 6–12%. Treated and raw samples were ground for 1 min with an electric grinder (Moulinette, Moulinex, Barcelona, Spain) and passed through a 150-µm sieve to ensure homogeneous particle size.

The apparent amylose content (AAC) was quantified using the method suggested by Juliano et al. [15]. A standard curve for the estimation of AAC (10 to 40 mg/100 mL of potato amylose) was used, and the absorbance was measured at 720 nm using a spectrophotometer (UH5300 UV/VIS Spectrophotometer, HITACHI Corp., Tokyo, Japan). The AAC of rice samples were expressed as mg/100 g dry matter (DM).

### 2.5. Microstructure by Scanning Electron Microscopy

Changes in the microstructure of pressurized rice samples were examined using scanning electron microscopy (SEM) imaging, according to the method described by Xu et al. [16]. Freeze-dried rice grain samples were mounted onto a SEM plate using conductive carbon tabs and Pt-coated via sputtering of 7 nm. The three-dimensional network microstructure of the rice grain samples was then observed and photographed using a JSM-5610-LV scanning electron microscope (JEOL Ltd., Tokyo, Japan) at a 20-kV acceleration voltage. For image analysis, the ImageJ2 v.1.54f image analysis software was used on the Java 8 platform [17]. Particle circularity was quantified with the method of Jordan et al. [18]. A circularity value of 1 is indicative of a perfect circle, while a value close to zero (0) suggests an elongated shape.

### 2.6. Pasting Properties

Flour samples (about 10% of moisture) were prepared as for the AAC determination (Section 2.3). Pasting properties of the rice flours were evaluated in a rotational Rheometer HAAKE RotoVisco 1 (Thermo Scientific, Karlsruhe, Germany) equipped with a cup Z43S and a starch blade FL2B paddle-shaped rotor with 2 blades using the method described by Kaur et al. [19] with slight modifications. Heating and cooling cycles were programmed. Each rice flour suspension (6 g/50 g) was held at 50 °C for 6 min, heated to 95 °C at 2.25 °C/min, held at 95 °C for 400 s, before cooling from 95 to 50 °C at 3 °C/min. The rotating speed of the paddle was set at 160 rpm during the measurements, except 960 rpm during the first 10 s. Device management and data acquisition were performed using HAAKE RheoWin 4 software (Thermo Scientific, Karlsruhe, Germany). Outputs were expressed as Pa·s.

### 2.7. Cooking Kinetics and Cooking Time Selection

To obtain the rice-cooking kinetics by boiling, 100 g of rice was submerged in 500 mL of osmotizedwater in a beaker and boiled (98 ± 1 °C) using a heating plate (Combiplac, JP Selecta, Barcelona, Spain). Subsamples of 15 g of rice were taken at two-minute intervals between minutes 6 and 14 and were placed on qualitative filter paper for 5 min. This process allowed for the removal of surface moisture from the rice grains.

For microwave-cooking kinetics, 60 g of raw rice was mixed with 300 mL of osmotized water and cooked using a domestic microwave oven (Taurus Luxus Grill 21 L, Taurus Group, Oliana, Spain) at 800 W. At two-minute intervals between 2 and 10 min, samples of 5 g of rice were collected for analysis and placed on filter paper, as previously described. The cooking time and textural properties for both cooking methods were determined by an instrumental analysis of the texture and a visual analysis of the grains. For each extraction time, samples were subjected to the texture profile analysis (TPA) as described in Section 2.9. Additionally, two grains of rice placed between two plates were crushed using a 5 kg weight load. The grains were analyzed under a magnifying glass, and the rice was identified as cooked when approximately less than 10% of the grains showed an opaque center [20].

### 2.8. Cooking Properties

Once the cooking time was determined for each cooking method, samples of cooked rice were prepared in accordance with the method outlined in Section 2.7, and the cooking properties were evaluated. Elongation ratio, gruel solid loss, water uptake ratio, and expansion volume were quantified according to the methods described by Bhattacharya et al. [21].

### 2.9. Instrumental Analysis of Texture Profile

The texture profile analysis (TPA) was performed with a texture analyzer TA-XT Plus (Stable Micro System Ltd., Surrey, UK), equipped with a 50 kg load cell and an aluminum compression platen (75 mm Ø, code P/75). The Exponent Lite v.6.1. software (Stable Micro Systems Ltd., Surrey, UK) was used. A double-compression test was programmed for TPA. A rice sample of 2 g (room temperature) was placed carefully on the base platform, under the center of probe, and compressed up to 90% at a test rate of 1 mm/s. Pre-test and post-test rates were 5 mm/s. Before analysis, the analyzer was calibrated using a 1 kg weight. Hardness (N), adhesiveness (N s), cohesiveness, resilience (%), springiness (%), gumminess (N), and chewiness (N) were evaluated applying the method described by Lyon et al. [22]. TPA tests were performed in triplicate.

### 2.10. Statistical Analysis

A one-way ANOVA was applied to data of amylose content and pasting properties. The HPP pretreatment × cooking time and HPP pretreatment × cooking method effects were evaluated using a two-way ANOVA. Differences between pairwise of means were tested via a Tukey’s test (95% significance level). The textural properties that were most affected by the dual treatments (HPP pretreatment and cooking method) applied to the rice in stage 2 were determined through a discriminant analysis. All the statistical analyses were carried out using SPSS Statistics for Windows, vers. 28.0 (IBM Corp., Armonk, NY, USA).

## 3. Results and Discussion

### 3.1. HPP

The pressure–time relation and the internal temperature of the sample recorded during rice HPP at 400 and 600 MPa are shown in Figure S1. The temperature of the filling water ranged from 25 to 30 °C during the treatments. The temperature of the product never exceeded 32 °C, with thermal leaps in the range of 8 to 10 °C. This means and ensures that the pressure treatment did not cause any heat-induced structural changes in the rice and starch and that any change in the properties, characteristics, and behavior of the samples should be attributed to the pressure.

### 3.2. Raw Grain Characterization and Water Uptake

The raw samples of *Nuovo Maratelli* rice have a length of 6.1 ± 0.4 mm and a length/width ratio of 2.12 ± 0.19, belonging to the medium grain classification [23].

The moisture content of *Nuovo Maratelli* samples was found to be 11.78 ± 0.53%, with an AAC of 22.61 ± 0.83% (25.31 ± 0.93 mg/100 g DM). Based on these findings, the samples can be classified as intermediate amylose rice [24]. Commonly, amylose content was positively related with hardness and negatively with stickiness, so cooked-rice hardness and stickiness were strongly and inversely correlated [25]. Although this *japonica* rice cultivar with intermediate content of amylose could be used to prepare *paella*, as HPP could reduce firmness and stickiness, the application of this technology could be of special interest to improve eating quality.

The soaking time at 30 °C necessary to reach the minimum recommended moisture content (from 25 to 35%) was determined to be 45 min.

### 3.3. Effect of Pressurization on Water Uptake Ratio

The water uptake ratios in the soaked and pressurized samples were 1.27 ± 0.00, 1.33 ± 0.02, and 1.69 ± 0.01 g treated/g initial rice for 0.1, 400, and 600 MPa, respectively. As a consequence, when comparing atmospheric pressure soaking to HPP soaking, the moisture content (g water/100 g) of normal rice grains significantly rose from 39.5 ± 0.45 (unpressurized samples) to 42.13 ± 0.17 (400 MPa) and 39.07 ± 1.51 (600 MPa). This is in accordance with the results described by Wu et al. [26], who found water absorption rates around 1.28 g/100 g in *Wuchang* cultivar, a non-waxy rice, after 400 MPa soaking for 15 min. The rise in moisture levels indicated that water molecules could effectively penetrate the peripheral regions of starch granules in rice grains under conditions of HPP [27]. The increase in weight with rising pressure levels did not correlate with a corresponding increase in moisture content. These findings suggest that solids retention was greater in samples subjected to 600 MPa, which is attributable to gelatinization processes that take place at these pressures [28,29].

### 3.4. Effect of Pressurization on Rice Grain Microstructure

**Figure 2:**
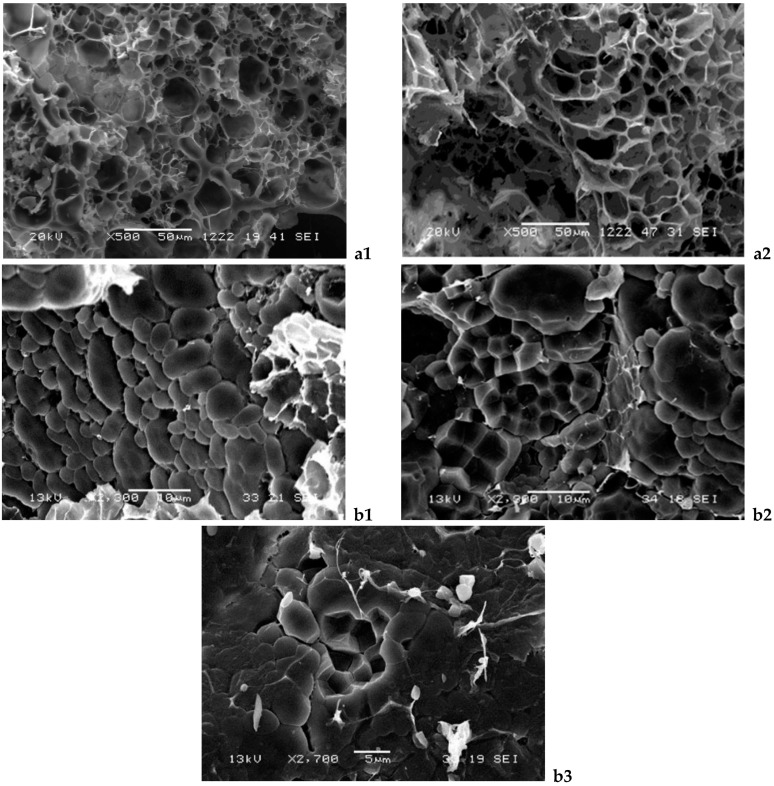
Scanning electron microscopy images of samples subjected to different treatments ((**a1**): raw rice; (**a2**): 400 MPa, ×500; (**b1**): raw rice; (**b2**): 400 MPa; (**b3**): 600 MPa, ×2300–2500).

Figure 2 depicts the alterations in the rice grain microstructure according to pressure treatments, as observed through SEM. The microstructure of rice grains was affected by HPP treatment. Moderate pressurization (400 MPa, 10 min) enhanced hydration of starch granules, making the cavities more homogenous (Figure 2(a1,a2)). The characteristic polyhedral forms of the starch of the raw rice grain (Figure 2(b1)) began to be lost after the application of 400 MPa (Figure 2(b2)). At 600 MPa, the microstructure underwent reorganization as a result of compaction and partial aggregation of the starch grains (Figure 2(b3)). Almeida et al. [12] observed similar changes in *japonica* rice starches treated at 400 and 600 MPa for 30 min. Seo et al. [11] pressurized *japonica* rice flours for 10 min and found that the starch structure was densest at 400 MPa and collapsed at 600 MPa. The pressurization favors the entry of water molecules into the granules, where they form new hydrogen bonds with the hydroxyl groups of the starch that replace the original intramolecular hydrogen bonds, leading to the destruction of the starch structure [30]. The intermediate amount of amylose could contribute to the stabilization of granules and the prevention of disintegrating, as found by Hu et al. [4] in non-waxy starch granules that retained their integrity, which is different from waxy starch granules that disintegrated after HPP at 600 MPa.

### 3.5. Effect of Pressurization on Pasting Properties of Rice Flours

The pasting properties of rice starch are influenced by granule swelling, amylose leaching, starch crystallinity, amylose content, and branch chain-length distribution of amylopectin. The increase in viscosity with rising temperature can be attributed to the removal of water from the exuded amylose by the granules as they undergo swelling [31]. Rice with a high–intermediate amylose content typically exhibits elevated values for pasting temperature (PT), peak viscosity (PV), minimum viscosity (MV), and final viscosity (FV). Additionally, it displays a low breakdown index (BI), indicating stability during the cooking process and a high setback, which means that it retrogrades easily [32,33].

The results of the assays with the *Nuovo Maratelli* rice showed no differences in any of the pasting properties in all the control and HPP-treated samples. However, the HPP-treated samples exhibited a tendency to increase PV at 400 MPa and to decrease this pasting parameter at 600 MPa, in comparison to the control (Table 1). These results are aligned with those previously reported by Li et al. [34], who applied pressures of 120–600 MPa to rice starch.

Modifications were observed in the microstructure of the starch granule at 400 MPa (Figure 2), this pressure being the threshold for rice grain gelatinization for the *japonica* cultivar *Chunyou* 84 [10]. All of this could explain the changes in the pasting properties of the 400 MPa pressurized sample compared to the control. On the contrary, under 600 MPa, the compaction and partial gelatinization of rice starch does not lead to changes in pasting properties respect to control.

In whole rice grains, the physicochemical modifications induced by HPP treatments which affect the pasting properties are less pronounced than in rice flour and rice starch; so, the effect on the pasting properties is therefore more challenging to assess.

### 3.6. Cooking Behavior: Cooking-Time Selection and Texture Changes

**Figure 3:**
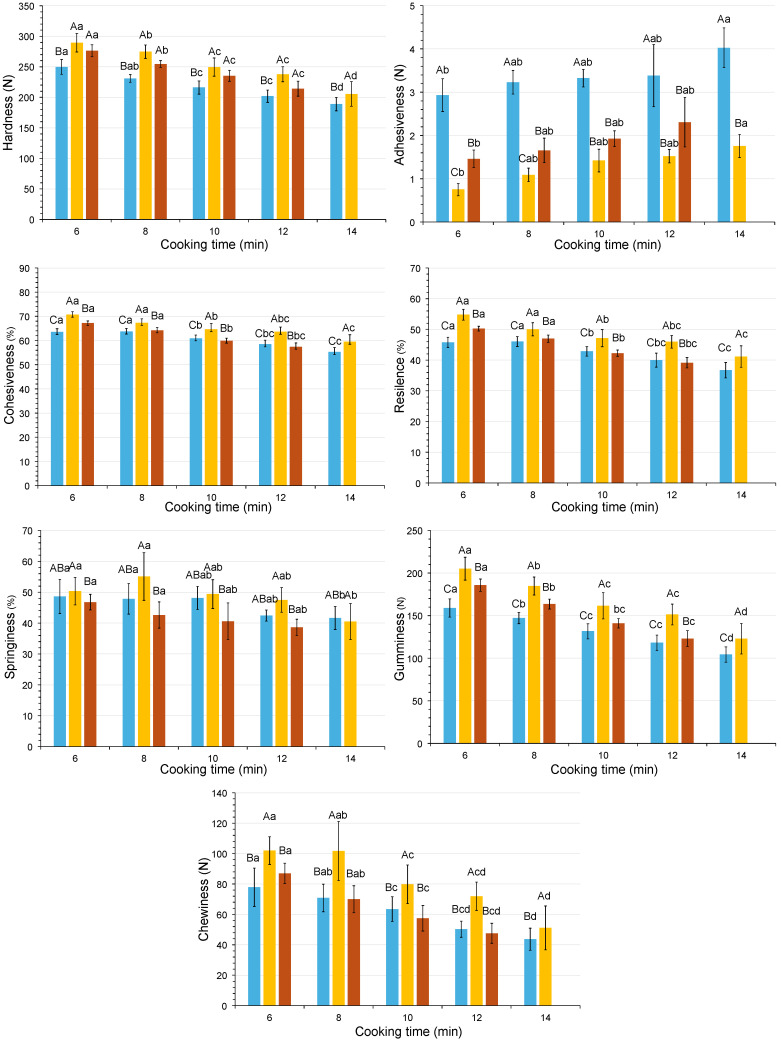
Kinetics of textural parameters measured by instrumental TPA, to establish the cooking time in rice samples treated by boil cooking (■: control; ■: 400 MPa; ■: 600 MPa). Note: Adhesiveness data are expressed as absolute values. The error bars indicate the standard deviation. Uppercase letters compare HPP pretreatments, and lowercase letters compare cooking times. Different letters represent significant differences via Tukey’s test (*n* = 3).

**Figure 4:**
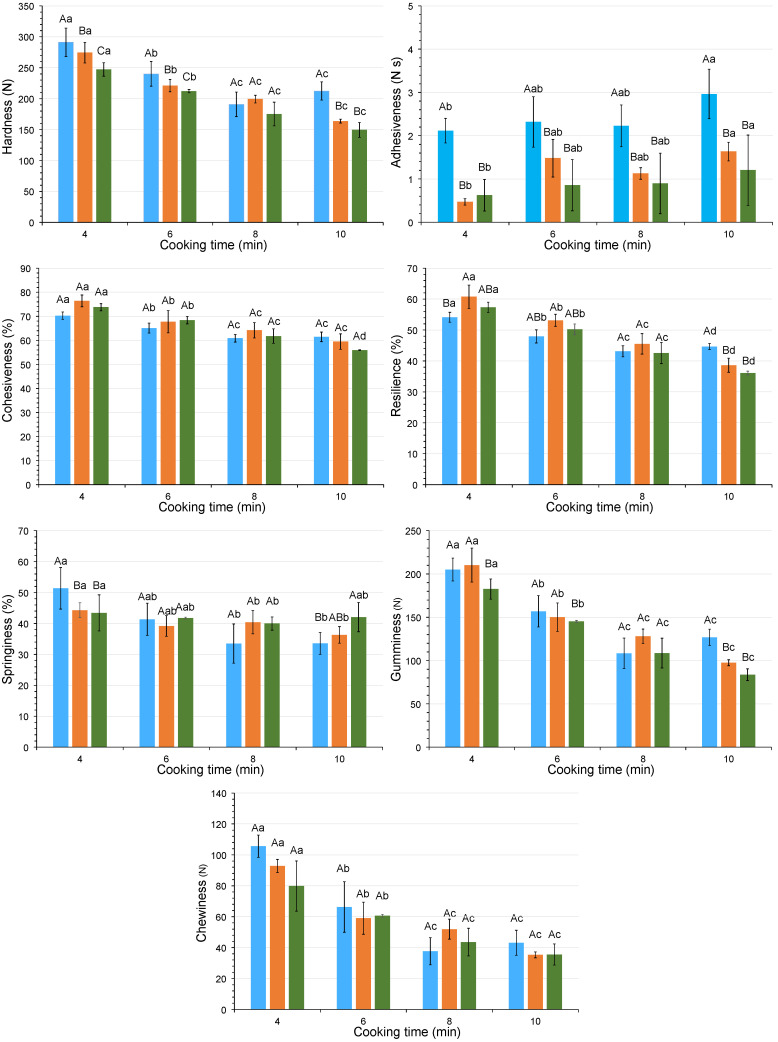
Kinetics of textural parameters measured by instrumental TPA to establish the cooking time in rice samples treated using microwave cooking (■: control; ■: 400 MPa; ■: 600 MPa). Note: Adhesiveness data are expressed as absolute values. The error bars indicate the standard deviation. Uppercase letters compare HPP pretreatments, and lowercase letters compare cooking times. Different letters represent significant differences via Tukey’s test (*n* = 3).

In this study, the cooking time was a prerequisite for evaluating the texture of the cooked rice. The changes in instrumental texture parameters as a function of HPP pretreatment and cooking time are shown in Figure 3 and Figure 4. Data for 600 MPa treated rice boiled for 14 min were not included because the samples were overcooked and were therefore not suitable for instrumental analysis. During cooking, all texture parameters decreased with time, except for adhesiveness, which increased. Overall, HPP treatment increased the cohesiveness and reduced the adhesiveness of the rice grains irrespective of the cooking method. However, the results of the texture kinetics showed that the effect of the other texture parameters depended on the cooking method applied. These effects were generally more pronounced in the 400 MPa than in the 600 MPa treated samples.

Compared to the unpressurized samples, the HPP samples showed a notable reduction in adhesiveness. In addition to changes in the amylose content in the cooked samples, which is negatively related to the stickiness, the structure of the starch affects this parameter [25], so the changes in adhesiveness could be explained by changes in the rice starch during pressurization. As the instrumentally measured adhesiveness is indicative of the stickiness between the rice grain and the TPA probe, it can be deduced that the leached materials (amylose) from the cooking process are a determining factor in the stickiness between the rice grains.

When boil cooking was applied (Figure 3), the pressurized samples had higher values for hardness, cohesiveness, chewiness, gumminess, and resilience than their non-pressurized counterparts at the same cooking time, especially when 400 MPa was used. In microwave-cooked samples, in contrast to boiling-cooked samples, HPP pretreatment reduced the hardness.

The more uniform structure shown in the 400 MPa pressurized rice than in control (Figure 2(a1,a2)) could be responsible for these changes. Tian et al. [27] also observed a more homogeneous network structure, resulting in lower hardness when normal and waxy rice were soaked at 400 MPa. In addition, the cooked rice also had smaller holes and a more resistant granule structure. Yu et al. [5] also reported that the hardness of HPP brown rice was reduced under pressure of 300 MPa for 10 min.

The cooking time was reduced when a pretreatment of 400 or 600 MPa was applied prior to cooking compared to the unpressurized samples. According to Priestley [35], fast-cooking rice is directly related to the water absorbed in the previous soaking. Since HPP favors water uptake through diffusion, it is expected that cooking time will be reduced. Thus, the application of intermediate pressures (around 400 MPa) induces starch gelatinization and may reduce cooking time by facilitating water diffusion into the rice grain [36,37]. In addition, when the pressure treatment is increased to 600 MPa, rice grain gelatinization occurs depending on the degree of the milling and/or treatment time [10], thus reducing cooking time.

Once the textural behavior of the samples subjected to the different treatments had been determined, it was necessary to fix the cooking times for rice processing in order to continue the research. According to the method described in Section 2.7, these times were therefore set at 14 and 10 min for the control samples cooked by boiling and microwave, respectively, 12 and 8 min for the 400 MPa HPP pretreated samples, and finally, 8 and 6 min for the 600 MPa HPP pre-treated samples. It should be noted that, although Billiris et al. [38] proposed instrumental texture as a method to establish the criteria for “well cooked rice”, they only quantified the maximum compression force by means of a one-cycle test.

### 3.7. Effect of HPP Pretreatment on Cooking Properties

To enable a comparison of the effect of the cooking method, the experiment was replicated with the cooking times selected in the previous section. The cooking properties of the raw rice samples (boiled and microwave cooked) and those treated with HPP are shown in Table 2. The soaking and pressurization processes caused water to penetrate the grain, increasing the moisture content of the soaked rice grains pressurized at 400–600 MPa, as indicated in Section 3.1. This pretreatment facilitated subsequent cooking by reducing cooking time, water uptake, and changes in grain physical dimensions. In addition, the cooking properties and impact of the HPP treatment differed depending on the cooking method. Thus, shorter cooking times were required for microwave cooking, although the application of the higher pressures reduced the difference between both methods. The partial gelatinization of starch by pressure at 600 MPa could explain this phenomenon [10].

As water uptake occurred during HPP and cooking time was reduced, lower ratios of these parameters were detected in the treated samples, especially at the higher pressure. Water absorption facilitates the incorporation of flavors into rice dishes, which is a crucial aspect from a culinary point of view. Similarly, the grain elongation ratio and the volume expansion after cooking in the HPP-treated samples were consequently smaller than those observed in the cooked non-pressurized samples. Furthermore, a lower final moisture content of the HPP-cooked samples was found, as already found in the uncooked pressurized samples, especially when cooked in the microwave. The physical changes in the grain dimensions during cooking were less pronounced at 600 MPa than at 400 MPa, probably due to a shorter cooking time and a partial pressure gelatinization before heat cooking. The compressibility of starch suspensions under pressure showed a reduction in total volume associated with gelatinization [39].

Furthermore, the effect of HPP treatments on the gruel solids loss differed depending on the cooking method. Microwave cooking increased the gruel solids loss, whereas boiling had no effect.

In terms of cooking properties, boiled rice showed superior convenience and quality compared to microwaved rice, which showed lower values for cooking properties, regardless of the pressure applied.

### 3.8. Effect of HPP Pretreatment on Textural Properties of Cooked Rice

The outputs of the instrumental texture evaluation for boiled and microwave-cooked rice grains are summarized in Table 3. According to the previous section, the cooking time was established as a function of the pressure and cooking method. The statistical analysis revealed that all parameters were significantly different between the unpressurized and pressurized samples, except for springiness (boiling and microwave cooking) and for resilience and chewiness (microwave cooking).

Regarding the hardness, the lowest value was observed in the unpressurized-and boil-cooked sample and the pretreated at 400 MPa plus microwave-cooked sample, whereas the highest was found in the pretreated at 600 MPa plus boiled sample. The lower hardness in pressurized-plus microwaved samples could be explained by a lower amylose leaching during cooking, which would form a film coating the rice grains [40].

Boiled pressurized samples displayed a higher hardness than control samples, while microwave cooked samples showed a reduced hardness, to a higher extent at 400 MPa pressure. A reduction in hardness was also detected for the oriental *japonica* rice treated at up to 300–500 MPa for 10 min [9]. Boluda-Aguilar et al. [41] found a decrease in instrumental hardness of *Jasmine* rice (an *indica* cultivar) heated by microwave in samples subjected to 300–400 MPa for 2–3 min, in comparison to untreated samples.

The pressurized samples, particularly those microwaved, showed lower adhesiveness than the unpressurized ones. Therefore, HPP pretreatment shortened the cooking time and modified the grain microstructure, affecting the adhesiveness. All the pressurized samples had more cohesiveness than the unpressurized and boiled sample, which showed the lowest value. A more compact network might be formed in rice under the high pressure that increases cohesiveness [27,42]. Similarly, differences in resilience only were found between the unpressurized and boiled sample and the other studied samples In the case of gumminess and chewiness, lower values were found in unpressurized and in 400 MPa treated and microwaved samples, whereas the highest value was observed in the 600 MPa pressurized and boiled sample. Moderate HPP treatment (up to 500 MPa) can cause the starch granules to be more firmly bound to the protein, thus decreasing the hardness of the rice and improving the gumminess and chewiness [26].

**Figure 5:**
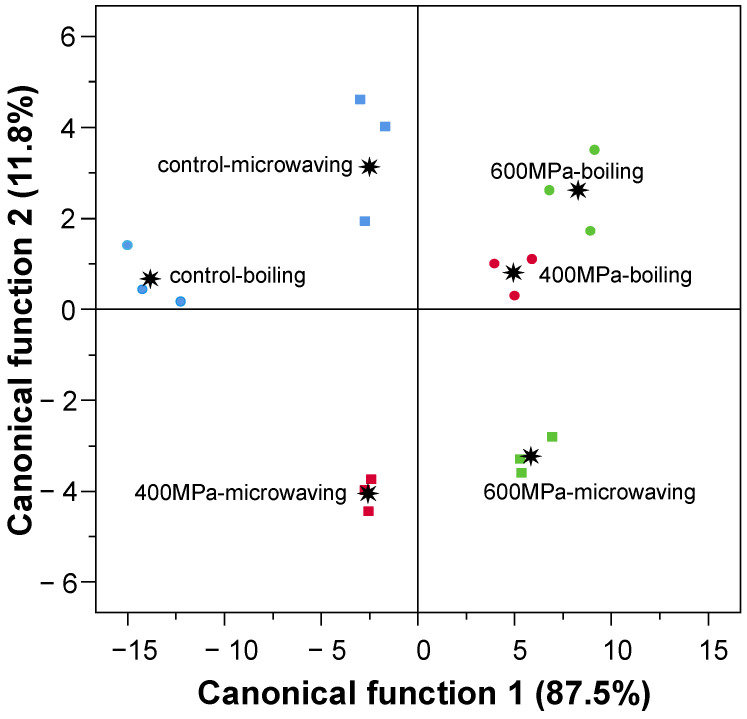
Score plot of the canonical discriminant functions for the instrumental texture evaluated in rice samples (✷: group centroids). blue: unpressurized; red: treated at 400 MPa; green: treated at 600 MPa; circle: boil-cooked; square: microwave-cooked.

The discriminant analysis performed to the instrumental texture parameters yielded two functions that collectively explained 99.2% of the sample variance. Discriminant function 1 (87.5%) was closely associated with hardness, gumminess, and resilience. Discriminant function 2 (11.8%) was strongly related to adhesiveness. Figure 5 depicts the samples based on the two discriminant functions. The unpressurized samples (control) are shown in the first quadrant of the graph. The pressurized and boiled samples are grouped in the second quadrant. The pressurized and microwaved samples are collected in the remaining quadrants.

In comparison to the unpressurized and boiled samples, the pressurized at 400 MPa and microwave-cooked samples showed comparable hardness values but had decreased adhesiveness. This finding suggests that microwave cooking had the greatest impact on the textural properties of pressurized samples, as hardness and stickiness are the key to improve and manage eating quality for rice [25].

### 3.9. Effect of Pressurizing and Cooking on Amylose Content

**Figure 6:**
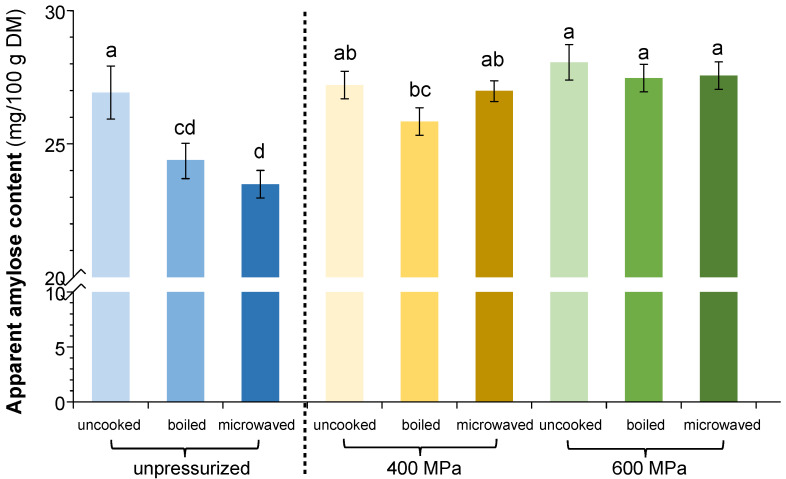
Changes in apparent amylose content in soaked-rice samples as a function of the treatments applied. The dashed vertical line separates unpressurized samples (**left**) from pressurized samples (**right**). Note the broken axis to highlight differences in treatments. Different letters indicate significant differences via Tukey’s test (*n* = 3).

Figure 6 shows the modifications in the AAC of rice samples soaked and subjected to the different treatments (pressurization and cooking). The highest values were recorded in the samples pressurized at 600 MPa, regardless of the cooking method (27.47–28.06 mg/100 g DM). The samples treated at 400 MPa displayed intermediate values (25.84–27.21 mg/100 g DM). Instead, soaked and microwaved samples showed the lowest values (23.49 mg/100 g DM).

Regarding the uncooked sample, the HPP pretreatment did not significantly impact the AAC. On the contrary, the cooked samples exhibited elevated AAC contents when subjected to HPP pretreatment, especially when microwave cooking was used and when the pressure applied was 600 MPa. Therefore, it can be concluded that pressurization would effectively reduce the leaching of amylose during the cooking process. This was also observed in pressurized normal and black rice grains [4,42], which was attributed to the intact starch granules presented in the HPP treatment that make amylose leaching difficult [4].

## 4. Conclusions

This study evaluates, for the first time, the effect of HPP pretreatment on the properties of *Nuovo Maratelli* rice cooked using two different cooking methods. The optimal cooking time was determined through the use of the TPA test. Based on the results obtained, pretreatments at 400 and 600 MPa for 10 min could be used to modify the cooking properties of the rice. In particular, the HPP treatment reduces cooking times and improves some of the textural properties that are critical for the eating quality of this rice cultivar. However, the shorten of the cooking time and the subsequent modifications depend on both the pressure level and the cooking method used. The application of the highest-pressure level facilitates the incorporation of water into the grain, thereby increasing its availability and reducing the cooking time by up to 6 min when microwaved. Additionally, the HPP pretreatment causes changes in the grain microstructure, which are already observed at 400 MPa and become more pronounced at 600 MPa, when a compact network was formed. Most of the textural parameters were modified as a result, but to improve the eating quality of the cooked rice, 400 MPa before microwave cooking could be selected, as it achieves a similar hardness to that of unpressurized-boiling-cooked rice and reduces adhesiveness. The results obtained in this work suggest that a pretreatment of pressurization could improve the eating quality and convenience of *Nuovo Maratelli* rice to prepare traditional rice dishes such as *paella*, making it more acceptable to consumers.

## Figures and Tables

**Table 1:** Pasting properties of *Nuovo Maratelli* rice flours as a function of pressurization treatment (10 min). Data are expressed as mean ± standard deviation (*n* = 3).

Parameter	Control	400 MPa	600 MPa	F Ratio	*p*-Value
PT	65.60 ± 1.82	66.30 ± 0.37	64.30 ± 1.29	2.752	0.142
PV (η/Pas)	24.10 ± 0.65 ^ab^	27.00 ± 2.32 ^a^	23.10 ± 1.11 ^b^	5.248	0.040
MV (η/Pas)	15.90 ± 0.95	18.30 ± 2.05	15.10 ± 1.17	3.855	0.084
FV (η/Pas)	33.90 ± 0.87	35.90 ± 2.47	34.00 ± 1.27	1.349	0.358
BI	7.90 ± 0.89	7.20 ± 1.04	7.40 ± 0.38	0.580	0.589

PT: pasting temperature; PV: viscosity maximum; MV: viscosity minimum; FV: final viscosity; BI: breakdown index. Different superscripts in each row indicate significant differences between means via Tukey’s test.

**Table 2:** Cooking properties of *Nuovo Maratelli* rice according to pressurization (10 min, 20 °C) and cooking treatments.

	Control		400 MPa		600 MPa	
Parameter	Boiling	Microwaving	Boiling	Microwaving	Boiling	Microwaving
Cooking time (min)	14 ± 0	10 ± 0	12 ± 0	8 ± 0	8 ± 0	6 ± 0
Moisture (g/100 g sample)	67.58 ± 0.06 ^a^	53.33 ± 0.53 ^d^	62.02 ± 0.91 ^b^	58.92 ± 0.35 ^c^	62.69 ± 0.25 ^b^	49.43 ± 0.77 ^e^
Water uptake ratio	3.21 ± 0.21 ^a^	2.42 ± 0.31 ^a^	1.90 ± 0.09 ^a^	1.36 ± 0.05 ^b^	1.39 ± 0.05 ^b^	1.28 ± 0.03 ^c^
Expansion volume	2.75 ± 0.28 ^a^	1.78 ± 0.31 ^b^	1.57 ± 0.09 ^bc^	1.36 ± 0.05 ^cd^	1.15 ± 0.04 ^cd^	1.12 ± 0.05 ^d^
Grain elongation ratio	68.50 ± 10.93 ^a^	60.48 ± 2.33 ^ab^	54.64 ± 2.51 ^bc^	46.99 ± 0.95 ^cd^	42.62 ± 1.64 ^cd^	41.59 ± 0.64 ^d^
Gruel solid loss (%)	3.83 ± 0.67 ^a^	1.28 ± 0.13 ^c^	3.81 ± 0.04 ^a^	1.77 ± 0.12 ^bc^	2.98 ± 0.85 ^ab^	2.32 ± 0.20 ^bc^

Different superscripts in each row indicate significant differences between means via Tukey’s test.

**Table 3:** Instrumental texture of unpressurized (control) and pressurized rice (10 min, 20 °C), cooked by the boiling or microwaving method (cooking time in Table 2). Data are expressed as mean ± standard deviations (*n* = 3).

	Control		400 MPa		600 MPa	
Parameter	Boiling	Microwaving	Boiling	Microwaving	Boiling	Microwaving
Hardness (N)	188.8 ± 11.0 ^c^	227.9 ± 16.1 ^ab^	237.8 ± 12.3 ^ab^	189.3 ± 11.6 ^c^	254.5 ± 5.5 ^a^	210.4 ± 7.1 ^bc^
Adhesiveness (N s)	−4.0 ± 0.5 ^c^	−3.4 ± 0.7 ^c^	−1.5 ± 0.2 ^b^	−1.1 ± 0.2 ^ab^	−1.6 ± 0.3 ^b^	−0.4 ± 0.1 ^a^
Cohesiveness (%)	55.2 ± 2.0 ^c^	62.2 ± 1.4 ^b^	63.6 ± 2.0 ^ab^	63.6± 4.1 ^ab^	64.2 ± 1.2 ^ab^	69.5 ± 1.0 ^a^
Resiliency (%)	36.73 ± 2.5 ^b^	45.0 ± 1.1 ^a^	45.9 ± 2.1 ^a^	45.3 ± 4.3 ^a^	47.0 ± 1.2 ^a^	51.5 ± 1.5 ^a^
Springiness (%)	41.7 ± 3.7	36.1 ± 3.4	47.5 ± 4.0	37.8 ± 2.1	42.6 ± 4.3	42.0 ± 6.7
Gumminess (N)	104.4 ± 9.0 ^c^	127.6 ± 9.1 ^bc^	151.4 ± 12.2 ^ab^	126.2 ± 15.2 ^bc^	163.5 ± 5.8 ^ab^	146.1 ± 6.7 ^a^
Chewiness (N)	43.7 ± 7.3 ^b^	47.7 ± 7.0 ^b^	72.0 ± 9.3 ^ab^	46.0 ± 4.8 ^b^	70.0 ± 8.9 ^a^	61.2 ± 5.1 ^ab^

Different superscripts in each row indicate significant differences between means via Tukey’s test.

## Data Availability

The original contributions presented in the study are included in the article and Supplementary Materials, further inquiries can be directed to the corresponding author.

## Supplementary Materials

The following supporting information can be downloaded at: https://www.mdpi.com/article/10.3390/foods13244052/s1, Figure S1: Pressure and temperature profiles recorded in the IDUS HPP 10L unit from rice samples: processing at 400 MPa (a); processing at 600 MPa (b).
