# Supplementary material for: Effect of High-Pressure Processing Pretreatment on the Textural Properties of Cooked Nuovo Maratelli Rice
Source: Foods. 2024 Dec 15;13(24):4052. doi: 10.3390/foods13244052 (PMC11727724; doi:10.3390/foods13244052)
Supplement: Supplementary file 1 [file foods-13-04052-s001.zip › foods-3344452-supplementary.pdf]

Effect of High-Pressure Processing Pretreatment on the Textural Properties of Cooked *Nuovo Maratelli* Rice

Cristina Arroqui, Sandra Horvitz, María José Noriega, Idoya Fernández-Pan, Francisco C. Ibañez and Paloma Vírseda

Supplementary material

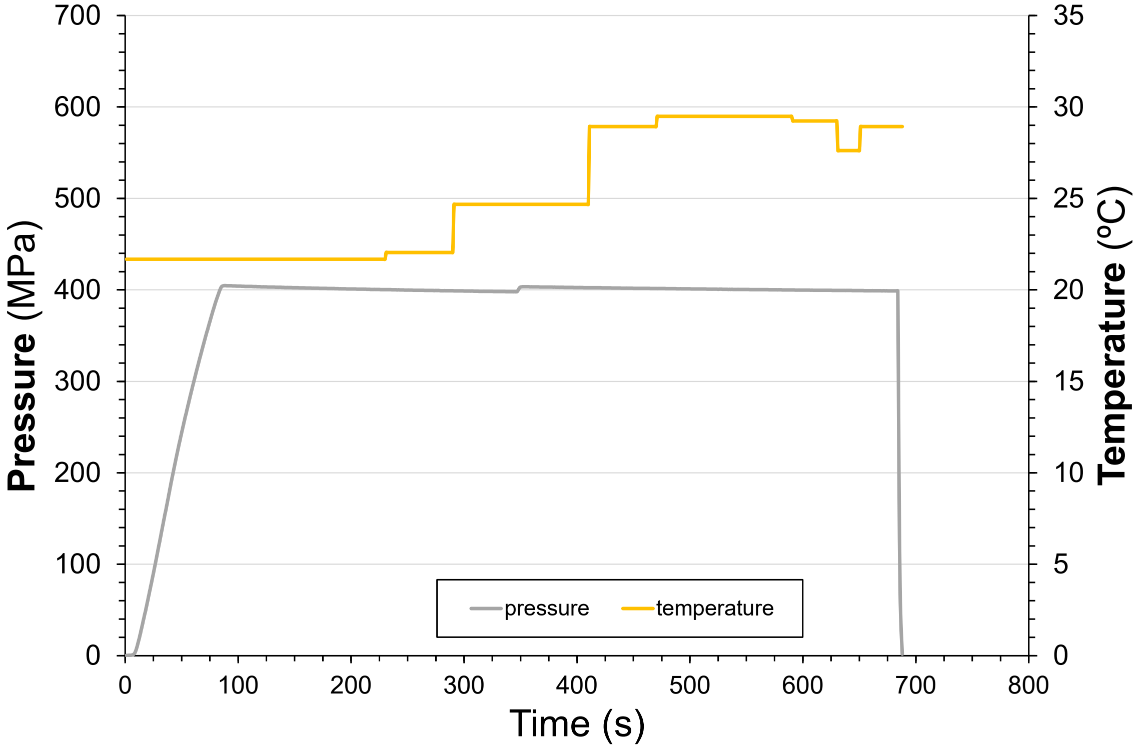

a

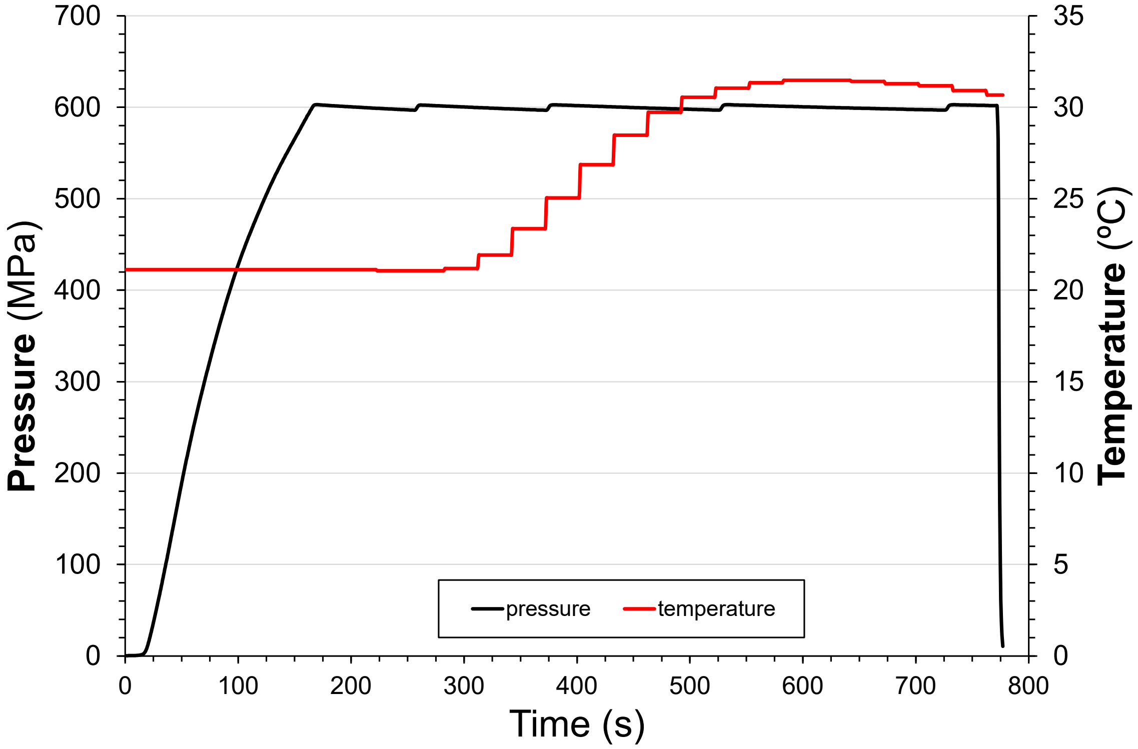

b

Figure S1. Pressure and temperature profiles recorded in the IDUS HPP 10L unit from rice samples: processing at 400 MPa (a); processing at 600 MPa (b).
